# Magnetic moment formation due to arsenic vacancies in LaFeAsO-derived superconductors

**DOI:** 10.1038/srep11280

**Published:** 2015-07-14

**Authors:** Konstantin Kikoin, Stefan-Ludwig Drechsler, Klaus Koepernik, Jiři Málek, Jeroen van den Brink

**Affiliations:** 1 School of Physics and Astronomy, Tel-Aviv University, 69978 Tel-Aviv, Israel; 2 Institute for Theoretical Solid State Physics, IFW-Dresden, P.O. Box 270116, D-01171 Dresden, Germany; 3 Institute of Physics, ASCR, Prague, Czech Republic; 4 Department of Physics, TU Dresden, D-01062 Dresden, Germany

## Abstract

Arsenic vacancies in LaFeAsO-derived superconductors are nominally non-magnetic defects. However, we find from a microscopic theory in terms of an appropriately modified Anderson-Wolff model that in their vicinity local magnetic moments form. They can arise because removing an arsenic atom breaks four strong, covalent bonds with the neighboring iron atoms. The moments emerging around an arsenic vacancy orient ferromagnetically and cause a substantial enhancement of the paramagnetic susceptibility in both the normal and superconducting state. The qualitative model description is supported by first principles band structure calculations of the As-vacancy related defect spectrum within a larger supercell.

The pronounced multiband character of the carrier spectrum is relevant to magnetic ordering and the unconventional Cooper pairing mechanism in Fe pnictides and chalcogenides^1^,^2^,^3^,^4^,^5^,^6^,^7^. Although certain features of strong correlation generated by the on-site Coulomb repulsion and local exchange (Hund’s rule coupling) in the Fe sublattice are suggested theoretically^8^,^9^ for strongly hole-doped Fe pnictides such as KFe_2_As_2_ and possibly evidenced experimentally^10^,^11^, it is generally believed that at least for optimally electron doped counter parts the basic features of electron-hole excitation spectra in these distinctly anisotropic materials may be revealed within a self-consistent band theory description. Nevertheless, inter-electron interaction between electrons in Fe(3*d*^*n*^) shells is significant, and this interaction is primarily responsible for the magnetic properties of these materials.

In particular, the magnetic response of iron pnictides substitutionally doped with non-magnetic impurities on the iron sublattice^12^ and structural defects such as As-vacancies^13^ demonstrate distinct features of localized magnetic moments arising on the background of the itinerant character of pristine materials. Such a behavior apparently shows evidence in favor of a near critical character of the electron-electron interaction between 3*d* electrons in iron pnictides. This interaction, however, is not strong enough to induce a Mott - Hubbard transition in the d-bands that form the Fermi surface of 1111 and 122 ferropnictides. However, a minor enhancement of the interaction in the vicinity of defect cells induces a magnetic response close to that of localized magnetic moments. The mechanism of such enhancement is discussed in this paper.

The general ideas of our theory have been schematically put forward in a brief preliminary communication^14^ based on the experimental observation of a strongly enhanced spin susceptibility^13^ pointing towards the formation of magnetic moments localized in the vicinity of As-vacancies (V_As_). Indications for the presence of static magnetic moments and enhanced paramagnetism in such As-deficient samples have also been found by *μ*SR-measurements^15^.

It was suggested that broken bonds around V_As_ (see Fig. 1) strongly distort the states around the hole pocket formed by *d*-electrons^13^,^14^. The related defect scattering can be strongly enhanced due to a singularity of the local Green function arising near the band edge. Here we expand upon and test the minimal model of Ref. 14 by comparing the basic assumptions used in the model with results of numerical calculations of the As-vacancy related electronic spectrum of As-deficient LaFeAsO. This spectrum is obtained from density functional theory based (DFT) supercell calculations and demonstrates the existence of two different localized states with different point symmetries. One of these states is a truly localized resonance deep in the filled bands, another state arises near the top of the hole band as anticipated in the minimal model. Therefore the DFT calculations substantiate this model, thus serving as a good starting point for further quantitative analysis of defect related properties of iron pnictides. We note that recently a similar Wannier-function calculation has been performed for Se-vacancies in monolayer^16^ FeSe but the magnetic aspects considered here have not been addressed.

The paper is built up as follows. We first introduce the minimal model for arsenic vacancies, as it is derived from the model Hamiltonian for iron-pnictides. Subsequently we establish the impurity scattering amplitudes at As-vacancies and the presence of localized states, which can be magnetic. We then present the first principles super cell calculations of As-vacancy related states in 1111 pnictides and present the numerical evidence for impurity-induced bound states. This is done by first introducing molecular orbital states on which the bands are projected and subsequently by the creation of the maximally projected Wannier functions for the Fe 3*d*, As 4*p* and O 2*p* orbitals. At the end of the paper our conclusions are presented.

## Minimal Model for Arsenic Vacancies

Various band calculations^3^,^17^,^18^,^19^,^20^,^21^ reveal the structure of valence and conduction bands which reflects the structure of covalent bonds in the As-Fe planes. Fe-related 3d orbitals form the top of the valence bands and the bottom of conduction bands, so that the d-partial waves form the hole and electron pockets of the Fermi surface. Two of 3d-orbitals, namely 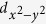 and 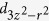 give main contributions to the lower part of the 3d-valence band, while the orbitals *d*_*zx*_ and *d*_*yz*_ dominate near its top. Three orbitals *d*_*zx*_, *d*_*yz*_ and *d*_*xy*_ form the electron pockets around the M points of the Brillouin zone. As-related *p*-partial waves contribute mainly to bonding (occupied) and antibonding (empty) bands well below and well above the d-waves around the Fermi level *ε*_*F*_. Such disposition reflects an essential role of *dp* covalent bonds in formation of the band spectra.

## Model Hamiltonian

The multiband Hamiltonian describing the band spectrum is


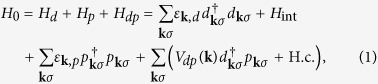


where the first three terms describe five Fe-related d-bands, three As-related p-bands and the interband hybridization responsible for the formation of occupied bonding and empty antibonding bands. The interaction term *H*_int_ which enters *H*_*d*_, contains both the on-site Coulomb - Hubbard repulsion *U* in the 3*d*-shells of Fe ions and the inter-orbital exchange.

The general structure of hybridized (*d*,*p*) bands is shown in Fig. 2. This picture schematically represents the results of our DFT calculations, which are presented in detail in the second half of this article. Having in mind the distribution of various *d* waves over the spectrum described above, and being interested mainly in the states around the Fermi surface formed by the holes near the top of the nearly fully occupied band around the Γ point in the Brillouin zone and the electrons in the electron pockets around the *M* points in its corners, we discuss in this section a minimal model. In this model only two pairs of orbitals are taken into account, namely *d*_*xz*,*yz*_ orbitals which dominate at the top of the nearly filled band *ε*_*k*,*d*_, and two *p*_*x*,*y*_ orbitals which are involved in the formation of broken bonds around the V_As_ centers.

**Figure 1:**
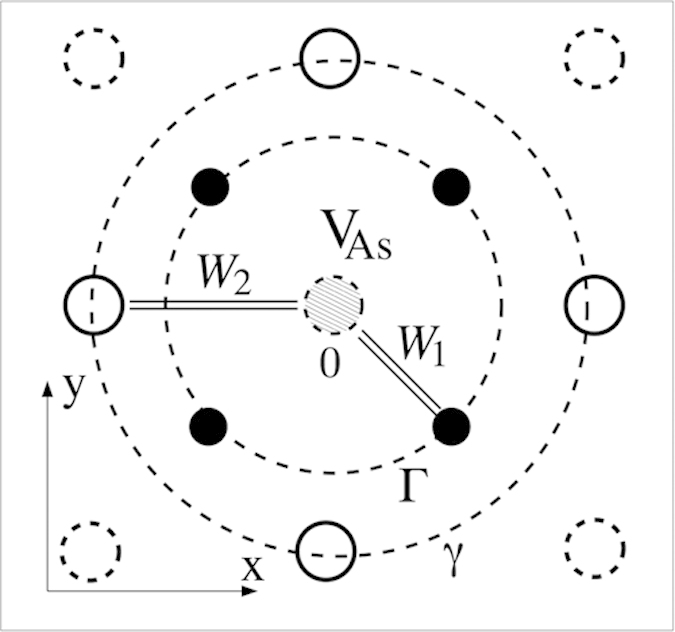
Schematic representation of bonds around a single arsenic vacancy V_As_ in a Fe-As layer. Fe ions: •. Arsenic ions above and below the Fe plane: solid and dashed ○, respectively. The *dp*-dangling bonds between the V_As_ and its four nearest Fe neighbors forming the orbital Γ are labeled as *W*_1_; the *pp*- dangling bonds between the central V_As_ orbital 0 and molecular orbital *γ* including four second neighbors in As tetrahedra are labeled as *W*_2_.

In accordance with the model of V_As_-related states^14^, the defect potential affects the *dp* hybridization and breaks the valence bonds in the As sublattice of layered iron pnictides. In addition, due to the large missing charge −3 *e* (within an ionic picture), the on-site energies of the surrounding atoms is changed and a relaxation of their positions takes place. The first effect is taken into account in electronic structure calculations that are presented later, whereas the latter one is ignored for the sake of simplicity. Figure 1 illustrated the structure of the broken bonds described by the defect potentials *W*_1_ and *W*_2_, respectively. The corresponding model Hamiltonian may be written in the following form:


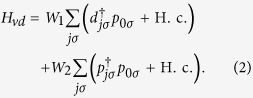


Here summation is performed over the sites *j* belonging to the first and second coordination spheres around the defect site 0, respectively in accordance with Fig. 1. The possible role of the electrons from the next coordination spheres is discussed briefly later on. Accepting this simple form for the defect Hamiltonian, we find an analytical solutions for *d*- and *p*-scattering amplitudes accounting for spin dependence induced by the interaction *H*_int_ between Fe-related d-electrons. Following the spirit of the basic models of local impurity moments in itinerant metals^22^,^23^, we retain only the Hubbard repulsion in this term


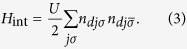


## Scattering Amplitudes and Localized States

To find the scattering amplitudes, one should solve the Lippmann - Schwinger equation


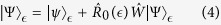


where |*ψ*〉 is the eigenvector for the unperturbed state described by the Hamiltonian *H*_0_, 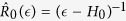 is the corresponding resolvent operator and 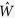 is the defect related perturbation described by the Hamiltonian Eq. (2).

**Figure 2:**
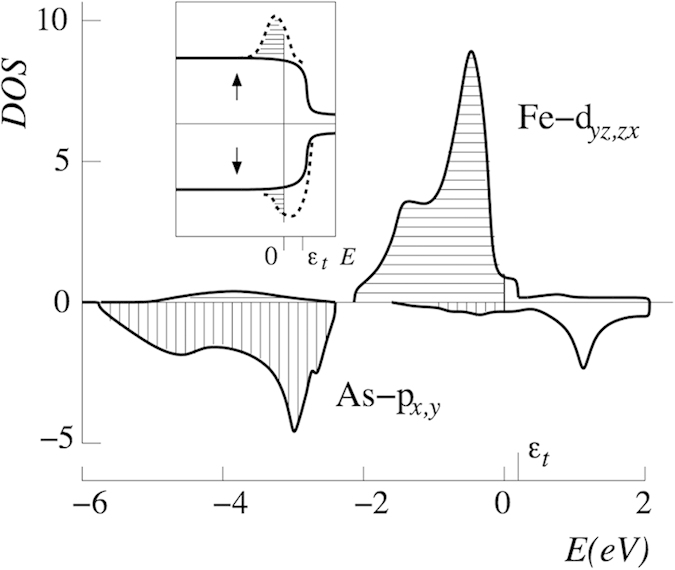
Partial DOS for Fe_*xz,yz*_ and As_*x,y*_ states, following the DFT-TB approximation of Ref. 19,20. *E* = 0 corresponds to *ε*_*F*_. Inset: vacancy induced peaks in the DOS near *ε*_*t*_ (the top of the hole *d*-band *ε*_*vb*_)

The operator equation Eq. (4) should be rewritten in a matrix form using the representation diagonalizing the Hamiltonian Eq. (1). Let us denote the three groups of eigenfunctions forming the DOS shown in Fig. 2 as |*va*〉,|*vb*〉,|*cb*〉. Here |*va*〉 represent the fully occupied band in the energy interval −6 eV < *ε* < −2.5 eV below *ε*_*F*_ = 0, |*vb*〉 are the states in the nearly filled band with a hole pockets at its top and |*cb*〉 are the states in the nearly empty conduction band forming the electron pockets at its bottom. In our minimal model the states |*va*〉, |*vc*〉 are represented mainly by *p*_*x*,*y*_ partial waves, and the states |*vb*〉 are predominantly *d*_*yz*,*zx*_ partial waves. The component *d*_*xy*_ is admixed to |*cb*〉.

In accordance with Fig. 1, the perturbation *W* is quasi local (only several coordination spheres are involved in formation of dangling bond states around V_As_). It is known that the integral equation Eq. (4) with a local kernel admits analytical solutions in the limiting cases; (i) local potential scattering^23^,^24^,^25^,^26^, resonance Breit-Wigner type scattering^22^,^27^,^28^ and combination of these two scattering mechanisms^29^ (see Ref. 30 for a general review of exactly solvable models for local scattering in non-metallic crystals). Our scattering mechanism cannot be reduced to either of these cases. Nevertheless, the analytic equations for the resonance states induced by the bond breaking perturbation Eq. (2) can be derived^14^.

**Figure 3:**
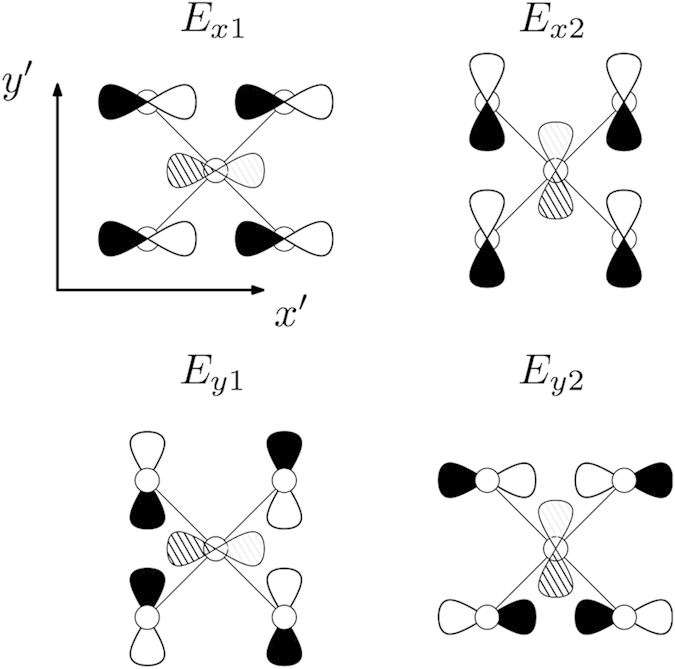
Irreducible 3d_(*xy*)*z*_ orbital combinations of *E* symmetry around the As-vacancy. The central orbital depicts a virtual vacancy 4*p*-orbital.

For this sake we introduce local “molecular” orbitals which obey the point symmetry of V_As_. This is the *C*_4*v*_ symmetry which decomposes the space of eight *d*_*yz*_, *d*_*zx*_ orbitals into the corresponding irreducible representations. These states *β* = |Γ*μ*〉 = |*E*_*xμ*_〉, |*E*_*yμ*_〉 are constructed and classified later on [see Fig. 3 and Eq. (18)]. Here *μ* = 1,2 stands for a row in the irreducible representation Γ. Similarly, basis states *α* = |*γν*〉 may be constructed for the orbitals 4*p*_*x*_, 4*p*_*y*_ centered at the As sites in the second coordination sphere. Together with the orbital centered at the central defect site these orbitals form the second subset *α* = 0, |*γν*〉

The Lippmann-Schwinger equation Eq. (4) may be rewritten in the local basis {*α*,*β*}. In this basis the perturbation operator *W* has the matrix elements *W*_1,*βα*_ and *W*_2,*αα*_′, which describe broken *d* − *p* and *p* − *p* valence bonds, respectively. In our minimal model only two pairs of *d* − type and *p* − type orbitals are taken into account. We are interested in local states centered at the defect site **R**_0_. Then the secular matrix for these states has the form


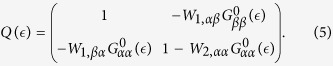


Here the cluster Green functions 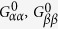 are obtained from the local lattice Green function


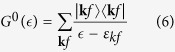


by means of projection operators


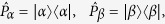


so that


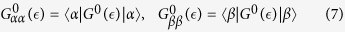


Index *f* = *vb*,*va*,*cb* denotes three bands involved in the formation of the local states around *ε*_*F*_ and in the depths of the continuum of valence states. In accordance with the partial wave analysis of the electron DOS we conclude that the projection operator 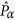 extracts mainly bonding and antibonding *p* states from the deep valence band and nearly empty conduction band, while 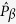 picks up mainly *d* states from the upper nearly filled valence band.

Such a structure of the partial DOS in a host quasi 2D crystal makes the electron spectrum extremely sensitive to broken bond defects^14^. Although the partial density of As-related *p* states is very small in the DOS around the Fermi level, the broken bonds violate the *pd* hybridization, and the dangling bond defect scattering induced by the potentials *W*_1,2_ in the secular matrix 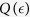 (5). This scattering results in the formation of localized states, however weak the scattering potential is, due to the nearly 2D character of the electron spectrum. Indeed, the 2D DOS has a step-like singularity near the top edges of all three bands. In accordance with general properties of the lattice Green functions^31^, these steps transform into logarithmic singularities around the edges *ε*_*tf*_ of the bands *ε*_*kf*_, namely


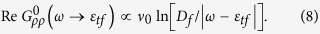


Here *ρ* = *α*,*β* and *D*_*f*_ is an effective bandwidth of the band *f*. The resonance states corresponding to the zeros of the function


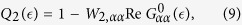


arise, in particular, above the top *ε*_*t*,*va*_ of the deep occupied band well below *ε*_*F*_.

Our main interest is however focused on the energy region around the Fermi level. At these energies the local Green function reaches its ultraviolet asymptotics, 
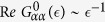
, so that 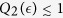, and the secular equation may be approximately written as


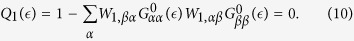


This means that the effective scattering potential in this region has the form


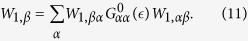


Thus Eq. (10) describes the defect induced distortion of the states near the edges of the band *ε*_*kvb*_. The energy dependence 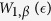 is smooth near the top of the band, but the local Green function 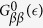 has a logarithmic singularity Eq. (8) in this region. As a result, Eq. (10) acquires resonance solutions in the vicinity of *ε*_*F*_^14^.

**Figure 4:**
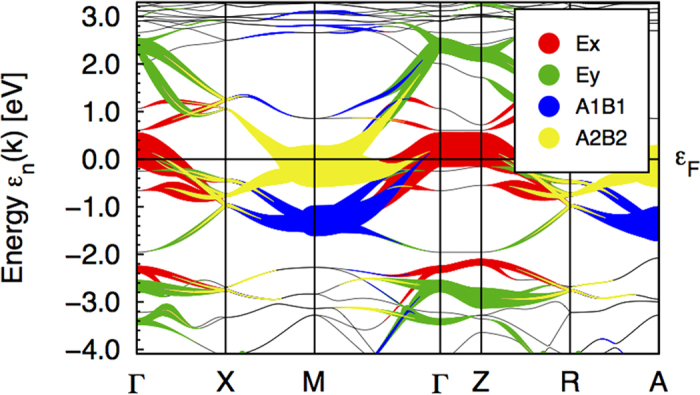
Molecular orbital projected bands for ordered LaOFeAs.

These resonance states correspond to the local orbitals |*β*〉 = |*E*_*xμ*_〉 (see Fig. 4), which are hybridized with their counterparts |*α*〉 with the same symmetry. Other molecular orbitals may form their own localized states due to scattering corresponding to singularities near the band edges in the depth of the valence bands. Our numerical analysis shows that such a resonance with the symmetry Γ = *A*_*g*_ arises near the bottom of the band *ε*_*kvb*_. The structure of the secular matrix describing the defect related states with *A*_*g*_ symmetry is similar to that in Eq. (5). In accordance with these calculation the wavefunction of this localized state is rather extended. This state is formed mainly by 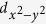 (and Fe 4*s*) orbitals. The role of the *dp*-hybridization in this case differs from that in the formation of near-edge states with *E*^*x*^ symmetry: broken *dp* bonds initiate bond breaking also between the 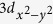 orbitals in the iron sublattice. We will return to the discussion of this difference later.

## Magnetic Solutions

Up to this point we ignored the spin structure of defect related resonance states. Now, we turn to magnetic properties of an arsenic vacancy V_As_. The vacancy itself is not magnetically active, but the effective scattering potential *W*_1,*β*_ Eq. (11) involves the molecular orbitals *β* = Γ*μ* enveloping four nearest iron neighbors (NN) and potentially even four Fe next-NN, as illustrated by the complex defect situation shown in Fig. 1. Then the orbitals *βσ* “inherit” the Coulomb repulsion from the 3*d* orbitals [see Eq. (3)]. This repulsion is the source of a spin-dependent scattering potential which is added to *W*_1,*β*_, namely


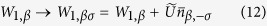


with


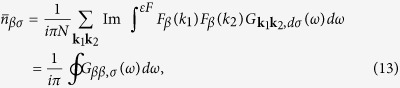


The integration contour envelops the poles of the full Green function *G*_*ββ*_ related to the resonance states near the top *ε*_*t*_ of the band *ε*_*vb*_. There are two orbitals 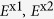 involved in the formation of the DOS near *ε*_*F*_ and two nearly degenerate sheets of hole Fermi surfaces. The defect related scattering potential preserves the point symmetry of V_As_, so the potential *W*_1_ is diagonal in *μ*. In our qualitative analysis we ignore the non-diagonal matrix elements of the Coulomb potential for the sake of simplicity. Then the orbital degeneracy gives a factor of 2 in the secular matrix, and this factor is included below in the definition of the local Green function. The corresponding residues are given by the poles of the defect related part of this function


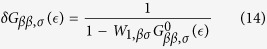


The Coulomb repulsion parameter 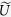 is renormalized due to *pd*-hybridization.


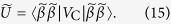


One expects that 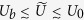, where *U*_*b*_ denotes the Coulomb repulsion for Fe in the bulk system without vacancies, due to the slight delocalization of the 3*d* wave functions because of *dp*-mixing and a reduced screening due to the missing arsenic atom^18^.

Inserting Eqn. (12),(14),(15) into Eq. (13), one transforms the latter equality into a pair of self-consistent equations


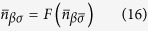


In accordance with the theory of defect related localized moments in metals^22^,^23^,^25^, these equations may have either only one trivial solution 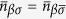, or three solutions, two of which correspond to the formation of a localized moment with 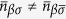. The necessary precondition for the formation of localized magnetic states in a system with itinerant electrons is the presence of an appreciable short-range spin-dependent interaction which may overcome the kinetic energy of the band electrons. Fe ions close to V_As_ serve as a source of such interactions due to the on-site Hubbard repulsion *U*_0_ in their 3*d* shells.

A magnetic solution arises when the repulsive potential exceeds some critical value^14^,^25^, namely 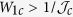, where


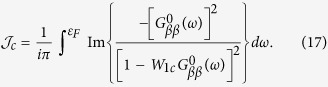


Such a magnetic solution is expected to be realized due to the logarithmic singularity of 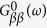 in the very vicinity of *ε*_*F*_. Due to this singularity in the denominator of the integrand in the r.h.s. of Eq. (17) the factor 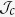 is strongly enhanced, favoring magnetic solutions. This implies that two defect-related narrow peaks arise in the DOS near *ε*_*F*_ (see the inset in Fig. 2).

To conclude this quantitative description, we emphasize that the minimal two band model is enough to describe the formation of a local magnetic moment around missing As atoms because the V_As_ potential Eq. (12) is effectively repulsive. Not only the Coulomb potential 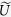 but also the hybridization induced term *W*_1,*βσ*_ is positive. It describes mutual repulsion of Fe related *β* states and As related *α* states. Since the center of gravity of the As_*x*,*y*_ partial DOS is below the center of gravity of the F*e*3*d*_*xz*,*yz*_ partial DOS (see Fig. 2), this repulsion shifts *β* levels upward. Thus we find that a nominally nonmagnetic arsenic vacancy is prone to induce a localized moment formed by states in the *h*-pocket due to the quasi-2D character of the electronic band spectrum in ferropnictides. As was pointed out in Ref. 14, the presence of such a local moment explains the observed strong enhancement of the magnetic susceptibility *χ*(0) in an As-deficient La-1111 system^13^.

## First Principles Super Cell Calculations

In order to substantiate the model of the previous sections, we performed density functional theory (DFT) calculations with the full potential local orbital code^32^ within the local density approximation^33^. We employed the scalar relativistic mode and a *k*-mesh of 4 × 4 × 6 in the whole BZ for the super cell described below. For the analysis in this section we focused on non-magnetic calculations, in order to keep the complexity as small as possible and to facilitate the extraction of disorder related states. Similar results are to be expected for magnetic calculations, since the results presented here are based on disorder effects only.

## Supercell and Molecular Orbitals

We created a supercell for LaOFeAs_1−*x*_, 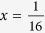 with a *c*-axis periodicity of one formula unit, which should be large enough since the *z*-direction Fe-Fe layer distance is larger than 8 *Å* and this compound does only show weak 3-dimensionality. The lateral supercell consists of 8 formula units in a square arrangement resulting in 16 Fe atoms. The As atom in the center is removed resulting in an As-vacancy V_As_, with a lateral V_As_-V_As_ distance of over 11 *Å*. We used the lattice constants *a*_0_ = 4.035 and *c*_0_ = 8.74 for the ordered unit cell and 
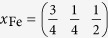
, 
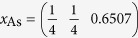
, 
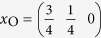
 and 
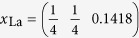
. We do not consider relaxation of the lattice around the As-vacancy as we do not expect qualitative changes to the results explained below.

The current analysis can be viewed complementary to a recent work^34^, who discussed the influence of As-vacancies in LaOFeAs on the band structure and magnetism. This work focuses at the magnetic state formation at different Fe sites and on the change of Fermi surface topology and density of state (DOS) changes due to the presence of a vacancy and local deformations. Though discussing impurity states they do not aim at identifying the character of such states.

Any localized impurity state sitting at the As-vacancy must contain a large weight of nearby orbitals. For the analysis we form molecular orbitals out of the 8 3*d*_(*xy*)*z*_ orbitals belonging to the four Fe neighbors and classify them according to irreducible representations of the local tetragonal *C*_4*v*_ symmetry. Some of these molecular orbitals form the hole pockets around the Γ-point (as shown below) and any relevant impurity state should also be formed of these orbitals, which have the strongest overlap with the As-vacancy site. We use a coordinate system, where the *x*,*y*-axes point along the Fe-Fe nearest neighbor directions and obtain a decomposition 8 3*d*_(*x*,*y*)*z*_ = *A*_1_ + *A*_2_ + *B*_1_ + *B*_2_ + 2*E*. Only the four *E* molecular orbitals couple with a virtual p-orbital at the vacancy site. If we assume orthogonality of the local 3d orbitals (assume Wannier orbitals) we obtain (Φ_(*xy*)*z*,*ab*_ being an orbital at an Fe site in (*a*,*b*)-direction from the vacancy site):


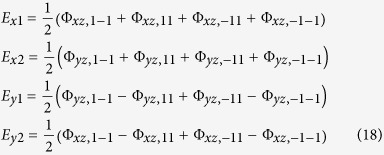


where *E*_(*xy*)1_ and *E*_(*xy*)2_ couple to 4*p*_*x*_ and 4*p*_*y*_ respectively, (see Fig. 3).

We can use these molecular orbitals instead of the local orbitals as projectors for the calculation of orbital projected band structures (“fat bands”). Such a construction gives an indication of the local symmetry of Bloch waves. We point out, that the total of such defined molecular orbitals does not necessarily form a basis. The projection is merely suitable for analyzing bonding patterns.

## Molecular Orbital Projected Bands

Figure 4 shows the fat bands according to the projection above for the ordered compound. As can clearly be seen the hole pocket around the Γ point is of *E*_*x*_ character, which also makes the van Hove singularity at the top of the hole band being formed of this combination of 3*d*_(*xy*)*z*_, while the electron pocket at the M-point has *A*_2_/*B*_2_ character. The *A*_1_/*B*_1_ combinations dominates around −1 eV at the M-point.

**Figure 5:**
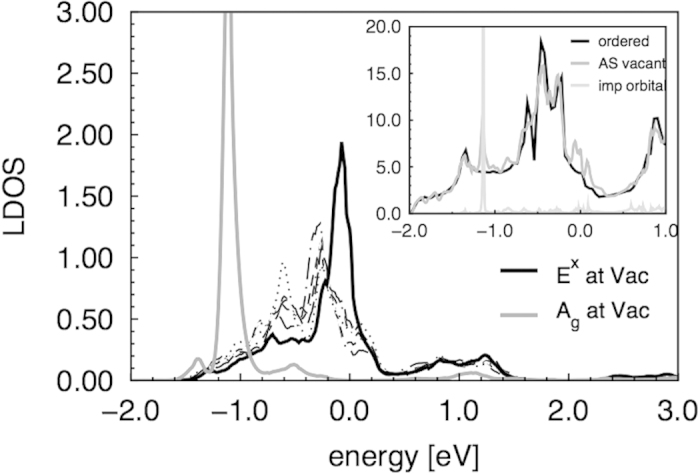
Molecular orbital projected LDOS. Black thick: *E*_*x*_ orbitals around the vacancy, Gray thick: *A*_*g*_ impurity state. Thin lines: *E*_*x*_ orbitals at other As sites. Inset: Black: total DOS ordered, medium grey: total DOS As-vacant, light grey: *A*_*g*_ impurity state empty orbital projection.

Figure 5 shows molecular orbital projected local densities of states (LDOS) for the As-vacant supercell. It shows the *E*_*x*_ projection around all As sites as well as the *E*_*x*_ and a new *A*_*g*_ projection around the vacancy. It can be noticed that the *E*_*x*_ LDOS around the vacancy is shifted towards the Fermi energy and has a pronounced peak at −80 meV compared to the *E*_*x*_ projections around the As sites, which are comparatively more dispersed. We read this as an indication of localization of wave functions with an *E*_*x*_ character around the vacancy. However, the still considerable band width would support an interpretation as impurity resonance rather than a truly localized impurity state. The second main feature at −1 eV below the Fermi level is an extremely narrow peak of *A*_*g*_ symmetry, which shows clear localization (small dispersion). This state is composed of a maximally symmetric combination of nearest neighbor Fe orbitals of 4*s* and 3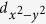 character in the ratio 2.5:1. The inset of Fig. 5 shows the total DOS of the ordered compound and the supercell and the DOS projected onto (manually introduced) empty site orbitals sitting at the vacancy, which proves the impurity nature and strong localization of this state. The picture also shows accumulation of spectral weight around the Fermi level as compared to the ordered DOS, which we mainly attribute to the *E*_*x*_ resonance states. Note that the LDOS of Ref. 34 shows both the *A*_*g*_ peak (although at a slightly lower energy for 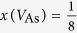) as well as the increase of the DOS near the Fermi energy.

## Maximally Projected Wannier Functions

To further support the interpretation above, we created maximally projected Wannier functions (WF) for the Fe 3*d*, As 4*p* and O 2*p* orbitals, which all show significant contributions to the occupied spectrum (and up to ~2 eV above the Fermi level). The WF creation includes the definition of an energy window, in which the Bloch states shall be represented by Wannier functions. If we do a simple band counting where we discard bands, without major contributions of these orbitals, we find that the relevant energy region of FeAs (and O) bands contains one more band than the number of Fe,As and O orbitals involved. This is a further indication that the *A*_*g*_ symmetry peak actually belongs to a well defined impurity state. In the creation of the maximally projected Wannier functions we use projectors onto suitably defined local orbitals, which for now are the corresponding local orbitals of our basis set and a single molecular orbital consisting of a symmetric combination of Fe 4*s* and 3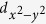 orbitals from the sites surrounding the vacancy in order to account for the additional band.

**Figure 6:**
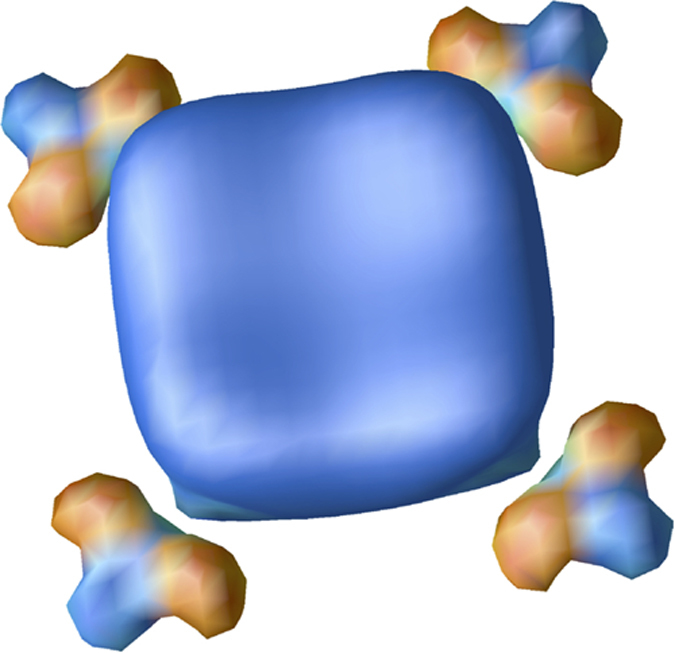
The *A*_*g*_ impurity state Wannier function. The As-vacancy sits at the center while the 3*d* lobes belong to the neighboring Fe atoms.

Figure 6 shows this *A*_*g*_ impurity state localized at the As-vacancy. Due to symmetry there is no 3*d*_(*xy*)*z*_ character in this states. Instead we find considerable La 4*d* weight from 8 *a*_*B*_ away and some As 4*p* weight from 6*a*_*B*_ away. The presence of Fe 4*s* weight is not surprising since the ordered bulk calculation shows that roughly 0.5 4 *s* electrons are occupied (strongly dispersed) and that the region around −1 eV shows some Fe 4*s* character. The impurity simply attracts these states. All resulting WFs are exponentially localized. The hopping integrals essentially fall off to zero after 15*a*_*B*_, although the hoppings for the WF of the *A*_*g*_ impurity state are a bit more far reaching (20*a*_*B*_) due to it consisting essentially of orbitals of 4 atoms. The resulting Wannier function fitted band structure has the property that the *A*_*g*_ impurity band contains 60% of the corresponding impurity state Wannier function, while the other 40% are due to other Wannier functions. The missing weight of the *A*_*g*_ WF is mostly spread over unoccupied bands between 0 and 2 eV. The fact that the impurity band is predominantly of impurity WF character supports the interpretation of a well defined localized *A*_*g*_ impurity state at −1 eV of the shape shown in Fig. 6. An important conclusion is that the impurity states is not made of a linear combination of 3d_(*xy*)*z*_ orbitals.

**Figure 7:**
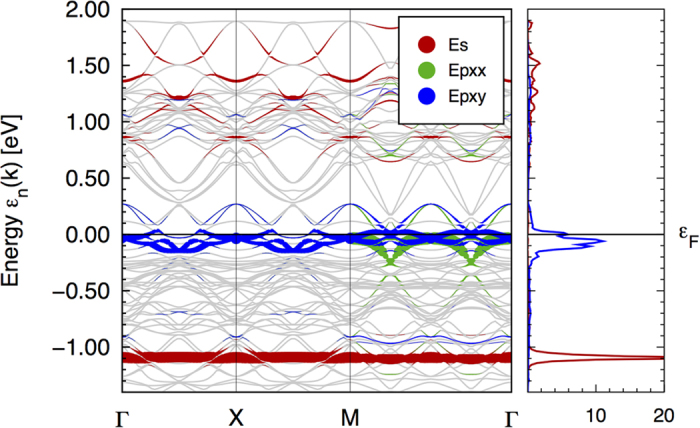
Wannier function bands and WF-LDOS of the *A*_*g*_ impurity state (light grey, red) and the *E*_*x*_ resonance (dark grey, blue).

In order to further elucidate the role of the latter orbitals in the state formation around the vacancy, we change the set of WF projectors in the following way. We replace the basis-orbital projectors for the 8 Fe 3*d*_(*xy*)*z*_ orbitals surrounding the vacancy by the irreducible combinations described above, add the *A*_*g*_ projector, the rest of the 3*d* orbitals at these 4 Fe sites and all basis-orbital projectors of all Fe sites not neighboring the As-vacancy. For simplicity we exclude the As and O orbitals, which is possible because of a gap between the As/O bands and the Fe bands at −2 eV. The resulting maximally projected WFs describe the Fe sector of the band structure excellently and exhibit the following features. All Wannier functions are exponentially localized. The *A*_*g*_ impurity state still has some weight above the Fermi level. The *E*_*x*_-doublet is well confined to the vicinity of the Fermi level, better even than the molecular orbital projection of Fig. 5. This is partially due to the fact that we can define an energy window, which provides the main contributions to a particular WF. This window can be narrowed as long as the essential weight of the resulting WF is confined to this window. This energy restriction does not work if the state is too dispersive and hence the fact that we can localize the *E*_*x*_ states in energy is a strong indication that these states form an impurity resonance. It is however, no strict impurity (as compared to the *A*_*g*_ band) as illustrated by Fig. 7 on the one hand due to its dispersion and due to the state counting argument presented above. In Fig. 7 we also depict the WF projected LDOS for the *A*_*g*_ impurity state and for the *E*_*x*_ resonance, which shows that the WF projected *E*_*x*_-LDOS is even better localized in energy than the molecular orbital projection based on the basis orbitals.

The analysis above thus shows that the As-vacancy gives rise to a well defined impurity state of Fe 4*s* and Fe 3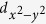 character at −1 eV below the Fermi level. This state is nearly non-dispersive and can robustly be visualized via molecular orbital projection, maximally localized Wannier functions and empty site orbital projection. We further demonstrated that the 8 Fe 3*d*_(*xy*)*z*_ orbital surrounding the vacancy form an impurity resonance of *E*_*x*_ symmetry, which is rather well localized in energy. These states are most likely important for the moment formation induced by the As-vacancy observed experimentally^13^,^15^.

An analysis for the slightly less anisotropic 122 parent system BaFe_2_As_2_ in its nonmagnetic state yields results similar to the ones reported above. Taken this together with the results reported in Ref. 40 for the related 11 Fe(Se,Te) systems, we strongly believe that the formation of localized states and the magnetic moment formation around As or Se(Te) vacancies is a common generic feature for all quasi-two dimensional iron pnictide or chalcogenide systems.

## Concluding remarks

We have elucidated the role of chemical bonds in formation of the resonance states (both magnetic and non-magnetic) induced by pnictogen vacancies in ferropnictides using As-deficient 1111 compound as an example. Qualitative analysis of the spectrum related to an isolated defect is supported by the numerical calculation of LDOS in a supercell approximation.

Of course, the supercell model considered may be only approximately mapped on the local defect model. The partial LDOS is nearly the same in both cases, but the logarithmic edge singularity Eq. (8) in the secular equation for the energy spectrum emerges only in the local model. However, the comparison of the two defect related states shows that unlike the truly localized state *A*_*g*_, the *E*_*x*_ LDOS is concentrated in the narrow energy interval around the top of the band *ε*_*vb*_. One may say that the defect related *E*_*x*_ states in a periodic supercell model “inherit” the singular features of the local lattice Green function 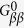. Thus we conclude that the numerical analysis presented substantiates the phenomenological approach with analytical solution. The latter solution, in turn, explains qualitatively the puzzling strong paramagnetic response of formally non-magnetic V_As_ defects.

The next puzzle is the relative insensitivity of the superconducting phase to these “magnetic” defects—actually *T*_*c*_ is even slightly enhancement by about 2 K^15^,^35^ which does not fit the prediction of a strong suppression of *T*_*c*_ by the conventional Abrikosov-Gor’kov theory of magnetic defects in effective single-band superconductors^36^. Recent calculations^37^,^38^ predict a similar destructive influence of conventional *non-magnetic* impurities on the *s*_±_ pairing mechanism in iron pnictides in general due to the related pair-breaking inter-band scattering. On the contrary, inter-band scattering by magnetic impurities is in this situation *not* pair-breaking^39^. It only causes some averaging (equalization) of the gaps on the hole and the electron-bands which are comparable from the very beginning. In this context a *s*_±_ based pairing mechanism seems to be favored at least for the title compound.

The slightly improved superconducting properties might be related to a suppression of remaining competing magnetic fluctuations coupled to the orbital fluctuations. Indeed, the Fe 3*d*_*xz*_ and *d*_*yz*_ orbitals involved in the formation of V_As_-defects are excluded from those fluctuations based on orbital splitting effects.

Thus, the As-vacancies stabilize the local tetragonal symmetry favorable for superconductivity and unfavorable for the competing magnetic stripe order. These rigorous but qualitative considerations as well as the studies of Refs. 34,40 make clear that a more advanced quantitative description of superconductivity in As deficient materials demands a more refined approach, which will be our future work. A microscopic theory for various defect states such as Zn, Ru, and other formally “nonmagnetic” substitutions at the Fe-sites remains a challenging task future systematic studies.

## Additional Information

**How to cite this article**: Kikoin, K. *et al*. Magnetic moment formation due to arsenic vacancies in LaFeAsO-derived superconductors. *Sci. Rep*. **5**, 11280; doi: 10.1038/srep11280 (2015).
